# Exploring the relationship between work–family conflict and sleep disturbance: a study on stratification and interaction

**DOI:** 10.3389/fpsyg.2023.1257233

**Published:** 2023-12-07

**Authors:** Jian Lee, Juyeon Oh, Heejoo Park, Juho Sim, Jongmin Lee, Yangwook Kim, Byungyoon Yun

**Affiliations:** ^1^Department of Health Administration, Yonsei University, Wonju-si, Republic of Korea; ^2^Department of Public Health, Graduate School, Yonsei University, Seoul, Republic of Korea; ^3^Department of Preventive Medicine, Yonsei University College of Medicine, Seoul, Republic of Korea; ^4^Department of Occupational Health, Graduate School of Public Health, Yonsei University, Seoul, Republic of Korea

**Keywords:** sleep disturbance, work–family conflict, working hours, quick return to work, engaged in large company, white-collar

## Abstract

**Introduction:**

Despite several studies on the association between work–family conflict (WFC) and sleep disturbances, a more comprehensive approach considering occupational factors is lacking. We aimed to analyze this association among Korean workers and the combined effects of WFC and job-related factors on sleep disturbance.

**Methods:**

Data on paid workers from the sixth Korean Working Conditions Survey were analyzed. Odds ratios (ORs) with 95% confidence intervals (CIs) for sleep disturbances with WFC were calculated using a multiple logistic regression model among Korean workers. Furthermore, stratification and interaction analyses were conducted between WFC and socioeconomic factors related to sleep disturbance.

**Results:**

Among the 24,923 workers (male: 11,752, female: 13,171) examined, 35.40% of males and 39.95% of females experienced sleep disturbances. In both sexes, the WFC group was significantly associated with sleep disturbance [adjusted OR (95% CI): male, 2.90 [2.67–3.16]; female, 2.54 [2.35–2.74]]. According to the stratification analysis, the association between sleep disturbance and WFC was prominent among younger and highly educated individuals, those engaged in quick returns, and larger companies in both sexes. In the interactions between WFC, quick return, occupation, and company size on sleep disturbance, there were significant additive associations, except between WFC and occupation among female workers.

**Conclusion:**

This study highlights the association between WFC and sleep disturbances in male and female workers and emphasizes the importance of maintaining work–life balance.

## 1 Introduction

Sleep disturbance is one of the most frequently reported health issues associated with several physical and mental health problems (Ramar et al., 2021; Joshi, 2022). Sleep disturbances in the short term has negative consequences, such as excessive daytime sleepiness (Shi et al., 2018) and reduced concentration (Sindi et al., 2018). Chronic sleep disturbance, characterized by short sleeping hours, increases the incidence of several diseases, such as cancer, migraine, diabetes, obesity, stroke, and cardiovascular disease (Itani et al., 2017; Ramar et al., 2021). It is also associated with a high risk of dementia (Shi et al., 2018), cognitive disorder (Sindi et al., 2018), and depressive symptoms (Sun et al., 2018).

Workers are particularly susceptible to sleep disturbance (Barnes and Watson, 2019), significantly influencing overall work efficiency. In the United States, the total economic burden of sleep disturbance care is estimated at $94.9 billion (Huyett and Bhattacharyya, 2021); in Australia, the figure is $7.7 billion (Streatfeild et al., 2021). Sleep disturbances among workers may result in several occupational disadvantages, including frequent absenteeism, workplace accidents, low job satisfaction and efficiency (Barnes and Watson, 2019), reduced productivity (Leitão et al., 2019), and slow job progression (Kucharczyk et al., 2012). Korean workers, who often work extended hours, face more severe sleep disturbances and shorter sleep durations than workers in other countries. According to an OECD report (OECD, 2013–2016), Korean workers dedicate considerable time to their jobs, ranking third regarding working hours among OECD countries. Simultaneously, they receive the least sleep, standing at the top of the list for the shortest sleep duration in the Organization for OECD and Development (OECD, 2018; Han and Kim, 2020).

In addition to sleep disturbance, work–life balance (WLB) plays a pivotal role in workers’ health. Defined as the balanced allocation of time and mental energy between professional and personal life while ensuring high satisfaction, WLB is crucial for psychological well-being (Greenhaus et al., 2003). WLB is closely related to various psychological health factors, including depressive symptoms (Hanson et al., 2014; Zhai et al., 2015), emotional exhaustion (Demerouti et al., 2004), sickness absenteeism, presenteeism, and fatigue (Choi and Kim, 2017). Poor WLB among Korean workers could be a critical issue compared to workers in other countries, ranking 36th among 38 OECD countries (Yang J. W. et al., 2018). One of the crucial triggers for poor WLB is work–family conflict (WFC; Vernia and Senen, 2022).

Some previous studies have found that WFC is deeply associated with sleep disturbance. Aazami et al. (2016) conducted a study examining how WFC affects sleep disturbances in Malaysian women who are part of the workforce, noting that the effect differs depending on age group. Buxton et al. (2016) found that higher WFC was associated with sleep sufficiency, poorer sleep quality, and more insomnia symptoms among individuals working in the field of information technology. Lallukka et al. (2010) analyzed the relationships between physical and psychosocial working conditions, work–family conflicts, and sleep disturbance among middle-aged employees in Helsinki, emphasizing the importance of addressing psychosocial stress and promoting work-life balance to reduce sleep disturbance and improve overall health among employees. Sato et al. (2021) highlighted the substantial burden of insomnia in Japanese working women in aged care services with elevated work–family conflict levels. However, there have been few studies that comprehensively investigate the relationship between WFC and sleep disturbance among both Korean representative male and female workers, considering diverse working conditions and potential confounding factors such as sociodemographic and socioeconomic variables. Given the relevance of sleep disturbance and WFC, a study that identifies groups at high risk for the relationship between sleep disturbance and WFC is necessary. Furthermore, there is a necessity to examine the additive interaction between WFC and other occupational factors on workers’ sleep disturbance, which has not been frequently discussed in previous studies.

Hence, this study aimed to investigate the association between WFC and sleep disturbance in a representative group of Korean workers. Moreover, we explored high-risk groups for sleep disturbance based on WFC to deepen insight into the growing relationship between WFC and sleep disturbance. We analyzed the combined effects of WFC and job-related factors on sleep disturbance. Through the identification of high-risk workers and effect modifiers with respect to the association between sleep disturbances and WFC, we hope that this study could be served as a basic data for establishing policies and guidelines to assist vulnerable workers in attaining a healthy sleep life.

## 2 Methods

### 2.1 Data and study design

**Figure 1:**
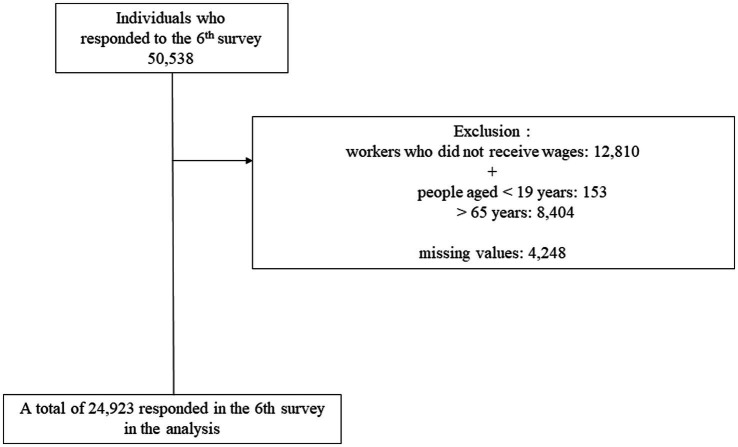
The schematic flow of the inclusion and exclusion criteria of participants.

We used data from the sixth Korean Working Condition Survey (KWCS) conducted by the Korean Occupational Safety and Health Research Agency in 2020. A total of 50,538 working individuals were surveyed and interviewed to ensure that the sample represented active Korean workers aged 15 years or older using a multi-area random sampling technique. All the respondents consented to participate in any additional scientific research and were de-identified. The validity and reliability of the KWCS were confirmed, and its quality was ensured using a well-designed random sampling process and a well-organized questionnaire (Kim et al., 2019; Min and Lee, 2021). For this study, 25,615 participants were excluded based on the following criteria: those who did not receive wages (*n* = 12,810), those aged <19 or > 65 years (*n* = 8,557), and those with missing values (*n* = 4,248). Finally, the data from 24,923 participants were included in this study. A schematic of the exclusion process is shown in Figure 1.

The study protocol adhered to the ethical principles of the 2013 Declaration of Helsinki and was approved by the Institutional Review Board of Severance Hospital (IRB: 4-2021-1046). The requirement for informed consent was waived owing to the retrospective nature of this study.

### 2.2 Outcome and independent variables

Our study utilized a set of three questions to ascertain the presence of sleep disturbance, while the assessment of WFC was conducted using five questions. Participants answered each question on a five-point Likert scale, and this scale was used with the scores reversed. We summed the scores to obtain the total score, with higher scores indicating more severe statements. Definitions of sleep disturbance and WFC are presented in Table 1. A minimal insomnia severity score (≥6) was used to define the presence of disturbance. A cut-off score of 6 for the sleep disturbance questions was deemed adequate to differentiate between those with and without sleep disturbances, with a sensitivity of 0.82 and specificity of 0.86, compared with the International Classification of Disease (ICD-10) research criteria for insomnia as a criterion standard in a population of 20–64 years (Broman et al., 2008). A cut-off of 11 for the definition of WFC was applied based on total scores ranging from 5 to 25. A total score of ≥11 refers to the presence of WFC (WFC group), while a total score of <11 refers to the absence of WFC (non-WFC group). With a Cronbach’s alpha value of 0.83, the scale’s five questions were internally consistent (An et al., 2020).

**Table 1:** The definition of the outcome and independent variables of the study subjects.

Variables		Questions	Answer	Definition
Outcome	Sleep disturbance	Within the last 12 months, how often have you had any of the following problems with sleep such as:	1. (never) 2. (almost never) 3. (sometimes) 4. (several times a week) 5. (all the time)	A total score of ≥6 was categorized as “sleep disturbance,” whereas a score of <6 was categorized as “non-sleep disturbance”
		i. Difficulty in falling asleep		
		ii. Waking up often while sleeping		
		iii. The state of being tired even after waking up		
Independent	WFC	Within the last 12 months, how often did you experience problems with:	1. (never) 2. (almost never) 3. (sometimes) 4. (several times a week) 5. (all the time)	A total score of ≥11 was categorized as “WFC,” whereas a score of <11 was categorized as “non–WFC”
		i. Kept worrying about work even when you were not working		
		ii. Felt too tired after work to do some of the household jobs which need to be done		
		iii. Found that your job prevented you from giving the time you wanted to your family		
		iv. Found it difficult to concentrate on your job because of your family responsibilities		
		v. Found that your family responsibilities prevented you from giving the time you should to your job		

### 2.3 Other covariates

Potential confounders and covariates included sociodemographic factors, such as age and sex, and socioeconomic factors, such as education level, average monthly income, and occupational classification. Age was categorized as <40 (young group) and ≥ 40 (old group) years based on life-transition period in Korea (Lim et al., 2013). An income of ≥2 million KRW (South Korean won) was categorized as the “high-income” group. Conversely, an income <2 million KRW was categorized as the “low income” group based on the median income in Korea [as of 2017] (Lee and Park, 2021). Educational level was classified into two groups: high school or below and university.

The occupational variables used as covariates included occupational classification, working hours per week or month, employment status, work duration, shift work, quick return, number of jobs, and company size. The occupational classification was categorized as white-collar (managers, professionals and related workers, and clerks) and other (craft and related trade workers, plants, machine operators and assemblers, elementary workers, service workers, sales workers, skilled agricultural, forestry, and fishery workers). Since a 40-h work week was enacted in Korea (Kang, 2021), working hours per week were categorized into ≤40 h per week and > 40 h per week. Employment status was segmented into “regular workers” and “non-regular workers,” with the latter comprising temporary and daily worker categories. Work duration was divided into ≥1 year and < 1 year. Shift work was split into the shift and non-shift work groups (using the question, “Do you work shifts?”). Quick returns were divided into quick and non-quick return groups (using the question, “Was there at least once last month that the interval from the end of work to the next day was less than 11 h?”). The number of jobs was classified into two groups: “one-job (1)” and “more-job (≥2).” Based on the number of employees, enterprise size was categorized as ≥300, <300, <50, and < 5 employees.

### 2.4 Statistical analysis

All analyses were performed by sex stratification. The baseline characteristics of the study population were compared using *t*-tests for continuous variables and chi-square tests for categorical variables according to WFC grouping. Using univariate and multiple logistic regression models, crude and adjusted odds ratios (ORs) and 95% confidence intervals (CIs) for sleep disturbance were estimated. We also conducted stratified analyses based on factors such as age [categorized as young (<40 years) and old (≥40 years)], education level, income, working hours, shift work, quick return, and occupational classification to examine the associations within these specific subgroups. Forest plots with ORs and 95% CIs were constructed for each outcome. *p* values were calculated for each stratum to determine the significance of the results. Moreover, interaction analyses of WFC with quick returns, company size, and occupation on sleep disturbance were conducted. The interaction was investigated using the relative excess risk due to interaction (RERI; Knol and VanderWeele, 2012) obtained using the delta method, regarded as a typical indicator of additive interactions.

Additionally, the study was conducted using the survey weight values assigned to each participant provided by the sixth KWCS to obtain unbiased estimators of the parameters. The sampling design weight estimated the survey weight, nonresponse rate adjustment weight, and poststratification adjustment weight (Cho and Kang, 2022). Furthermore, WFC status was divided into quartiles based on the overall scores and named as low WFC (<25%), low-middle WFC (<50%), high-middle WFC (<75%), and high WFC (≥75%). Value of *p* < 0.05 was considered statistically significant for all analyses in all two-tailed statistical tests. All statistical analyses were performed using the R software (R Foundation for Statistical Computing, Vienna, Austria, version 4.2.1).

## 3 Results

The basic occupational characteristics of male and female workers are summarized in Table 2. Among the 24,923 participants, 11,752 (47.15%) were male (mean age 44.8 ± 10.7 years). Further, 4,164 (35.40%) male and 5,262 (39.95%) female reported sleep disturbances. Male and female workers with sleep disturbance were older, worked >40 h per week, had higher education levels, received higher income, engaged in long working hours, had long work durations, had quick returns, were white-collar workers, and worked for large companies (*p* < 0.05). Table 3 summarizes the estimated ORs and 95% CIs of sleep disturbance according to WFC in the final model using multiple logistic regression analysis. The fully adjusted OR (95% CI) of sleep disturbance with WFC in male workers was (2.90 [2.67–3.16]; *p* < 0.001), and the full OR in female workers was (2.54 [2.35–2.74]; *p* < 0.001).

**Table 2:** Baseline characteristics of participants by sleep disturbance.

	Male			Female		
Variable	Non sleep disturbance (*n* = 7,588)	Sleep disturbance (*n* = 4,164)	*p* value	Non sleep disturbance (*n* = 7,909)	Sleep disturbance (*n* = 5,262)	*p* value
Age			<0.001			<0.001
Mean (SD)	41.3 (11.3)	44.8 (10.7)		43.0 (11.6)	45.9 (11.0)	
WFC			<0.001			<0.001
No (non–WFC)	5,862 (72.6%)	2,216 (27.4%)		5,919 (67.7%)	2,824 (32.3%)	
Yes (WFC)	1,726 (47.0%)	1,948 (53.0%)		1,990 (44.9%)	2,438 (55.1%)	
Education level			<0.001			<0.001
University or more	2,638 (61.9%)	1,623 (38.1%)		3,225 (57.2%)	2,411 (42.8%)	
High school	4,950 (66.1%)	2,541 (33.9%)		4,684 (62.2%)	2,851 (37.8%)	
Income (2 million KRW)			0.008			0.002
Low income	1,300 (67.2%)	634 (32.8%)		3,969 (61.4%)	2,494 (38.6%)	
High income	6,288 (64.0%)	3,530 (36.0%)		3,940 (58.7%)	2,768 (41.3%)	
The number of jobs			1.000			0.370
One-job	7,552 (64.6%)	4,145 (35.4%)		7,874 (60.1%)	5,232 (39.9%)	
More-job	36 (65.5%)	19 (34.5%)		35 (53.8%)	30 (46.2%)	
Working hours			<0.001			0.012
≤40 times	5,127 (65.8%)	2,666 (34.2%)		5,960 (60.7%)	3,862 (39.3%)	
>40 times	2,461 (62.2%)	1,498 (37.8%)		1,949 (58.2%)	1,400 (41.8%)	
Work duration			0.097			0.044
<1 years	1,050 (66.5%)	530 (33.5%)		1,365 (62.0%)	837 (38.0%)	
≥1 years	6,538 (64.3%)	3,634 (35.7%)		6,544 (59.7%)	4,425 (40.3%)	
Shift work			0.070			0.119
Non shift work	6,844 (64.8%)	3,711 (35.2%)		7,272 (60.3%)	4,797 (39.7%)	
Shift work	744 (62.2%)	453 (37.8%)		637 (57.8%)	465 (42.2%)	
Quick return			<0.001			<0.001
Non quick return	7,293 (66.1%)	3,740 (33.9%)		7,775 (60.7%)	5,043 (39.3%)	
Quick return	295 (41.0%)	424 (59.0%)		134 (38.0%)	219 (62.0%)	
Employment status			0.349			0.701
Non regular worker	1,112 (65.6%)	583 (34.4%)		1,450 (60.4%)	950 (39.6%)	
Regular worker	6,476 (64.4%)	3,581 (35.6%)		6,459 (60.0%)	4,312 (40.0%)	
Occupational classification			0.365			<0.001
Others	4,102 (64.9%)	2,214 (35.1%)		3,760 (58.0%)	2,718 (42.0%)	
White–collar	3,486 (64.1%)	1,950 (35.9%)		4,149 (62.0%)	2,544 (38.0%)	
Enterprise size			<0.001			<0.001
<5	1,395 (69.4%)	614 (30.6%)		2,365 (64.1%)	1,322 (35.9%)	
<50	3,521 (64.0%)	1,979 (36.0%)		3,952 (60.6%)	2,574 (39.4%)	
<300	1,631 (63.9%)	920 (36.1%)		1,239 (54.8%)	1,022 (45.2%)	
≥300	1,041 (61.5%)	651 (38.5%)		353 (50.6%)	344 (49.4%)	

Values are expressed by *n* (%) or mean (SD).

**Table 3:** The ORs (95% CIs) of sleep disturbance by WFC in logistic regression model.

Sex	WFC	Crude model			Model 1			Model 2		
		Odds ratios	CI	*p*	Odds ratios	CI	*p*	Odds ratios	CI	*p*
Male	No	1.00 (reference)			1.00 (reference)			1.00 (reference)		
	Yes	2.99	2.75–3.28	<0.001	3.02	2.78–3.28	<0.001	2.90	2.67–3.16	<0.001
Female	No	1.00 (reference)			1.00 (reference)			1.00 (reference)		
	Yes	2.57	2.38–2.77	<0.001	2.59	2.40–2.79	<0.001	2.54	2.35–2.74	<0.001

Model 1: adjusted by age, education, and income. Model 2: adjusted by age, the education level, income, the number of job, working hours, work duration, shift work, quick return, employment status, occupation, and company size.

**Figure 2:**
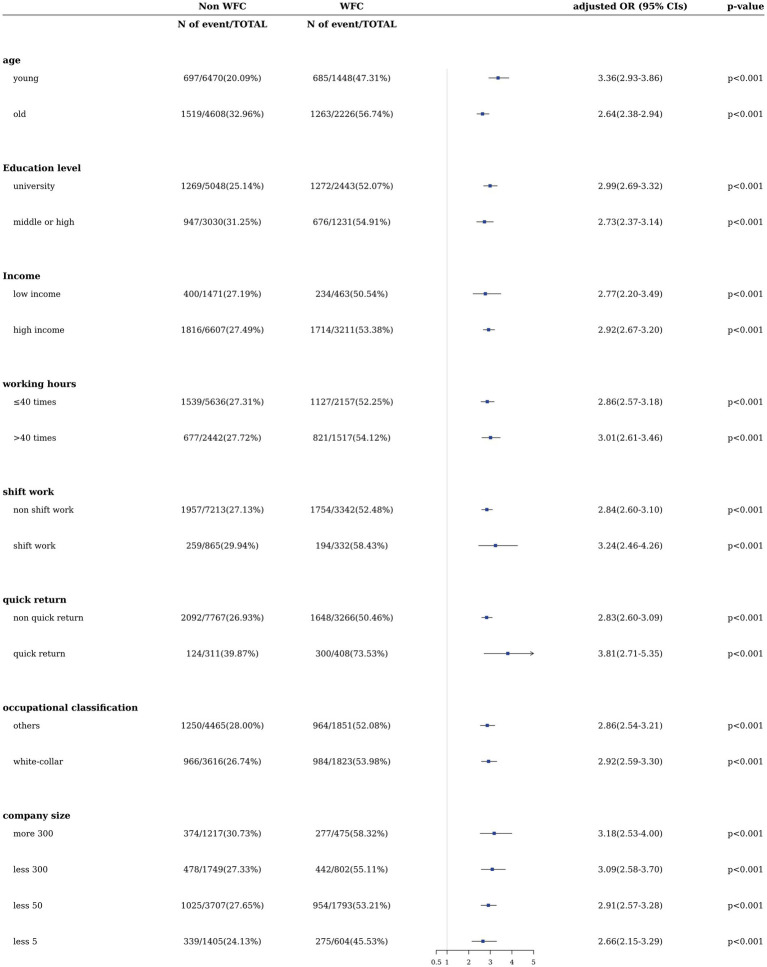
Adjusted ORs (95% CIs) of sleep disturbance by WFC among subgroups in male workers.

**Figure 3:**
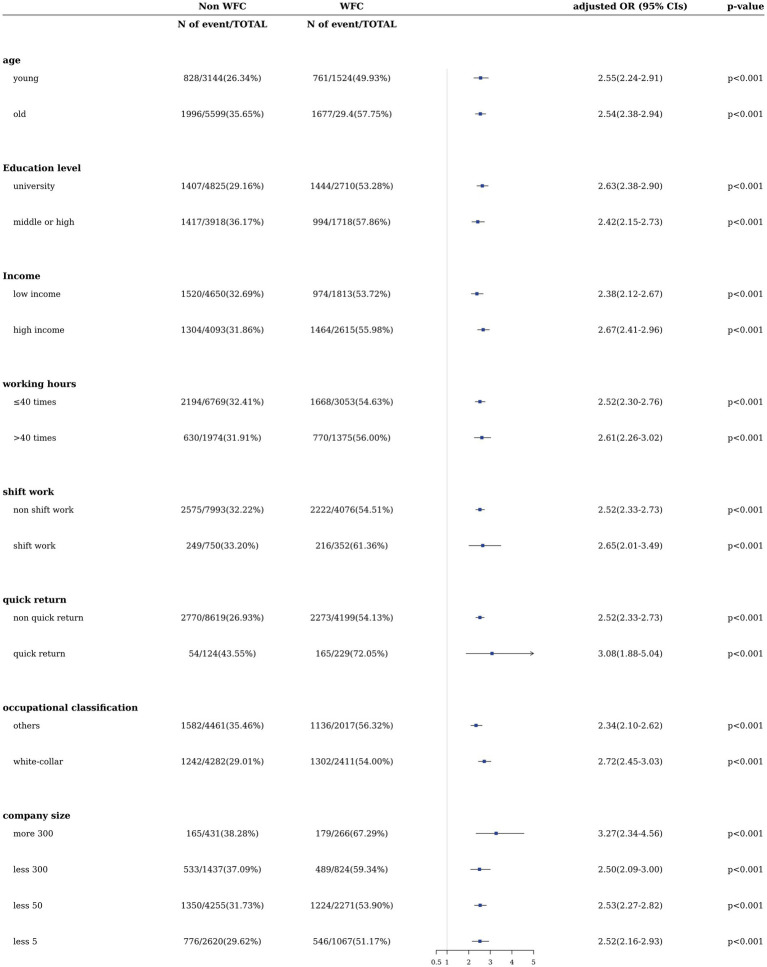
Adjusted ORs (95% CIs) of sleep disturbance by WFC among subgroups in female workers.

The full ORs (95% CI) of sleep disturbance according to WFC for each covariate stratification in male and female workers are shown in Figures 2, 3, respectively. All fully adjusted ORs for sleep disturbance according to WFC were statistically significant. For male workers, the association between sleep disturbance and WFC was prominent among younger and highly educated workers engaged in quick returns and larger companies in male and female workers (Figures 2, 3). Table 4 shows the significant results of the synergistic effects of simultaneously WFC and quick return, and company size and occupation on sleep disturbance, except for the additive association between WFC and occupation among female workers. RERI value for quick return and WFC on sleep disturbance was prominent among male workers (4.13 [2.35–5.91]; *p* < 0.001), and that for company size and WFC on sleep disturbance was prominent among female workers (1.89 [0.58–3.20]; *p* = 0.002).

**Table 4:** The ORs (95% CI) of sleep disturbance by interaction variables between **(A)** Quick return/**(B)** Occupation and WFC.

		*N*/Total		OR (95% CI)		Measure of interaction on additive scale: RERI (95% CI)
(A)	WFC	Non–quick return	Quick return	Non–quick return	Quick return	
Male	No	2,092/7,767 (26.93%)	124/311 (39.87%)	1.00 (reference)	1.73(1.36–2.01); *p* < 0.001	4.13 (2.35–5.91); *p* < 0.001
	Yes	1,648/3,266 (50.46%)	300/408 (73.53%)	2.81(2.57–3.06); *p* < 0.001	7.77 (6.17–9.79); *p* < 0.001	
Female	No	2,770/8,619 (32.14%)	54/124 (43.55%)	1.00 (reference)	1.50 (1.03–2.15); *p* = 0.033	2.37 (0.71–4.03); *p* = 0.003
	Yes	2,273/4,199 (54.13%)	165/229 (72.05%)	2.51 (2.33–2.72); *p* < 0.001	5.40 (4.01–7.27); *p* < 0.001	
(B)	WFC	*N*/Total		OR (95% CI)		Measure of interaction on additive scale: RERI (95% CI)
		Others	White-collar	Others	White-collar	
Male	No	1,250/4,465 (28%)	966/3,613 (26.74%)	1.00 (reference)	1.06 (0.94–1.19); *p* = 0.334	0.51 (0.07–0.95); *p* = 0.011
	Yes	964/1,851 (52.08%)	984/1,823 (53.98%)	2.80 (2.49–3.13); *p* < 0.001	3.21 (2.82–3.65); *p* < 0.001	
Female	No	1,582/4,461 (35.46%)	1,242/4,282 (29.01%)	1.00 (reference)	0.86 (0.77–0.96); *p* = 0.006	0.19 (−0.12–0.50); *p* = 0.114
	Yes	1,136/2,017 (56.32%)	1,302/2,411 (54.00%)	2.30 (2.06–2.57); *p* < 0.001	2.38 (2.10–2.69); *p* < 0.001	

**Figure 4:**
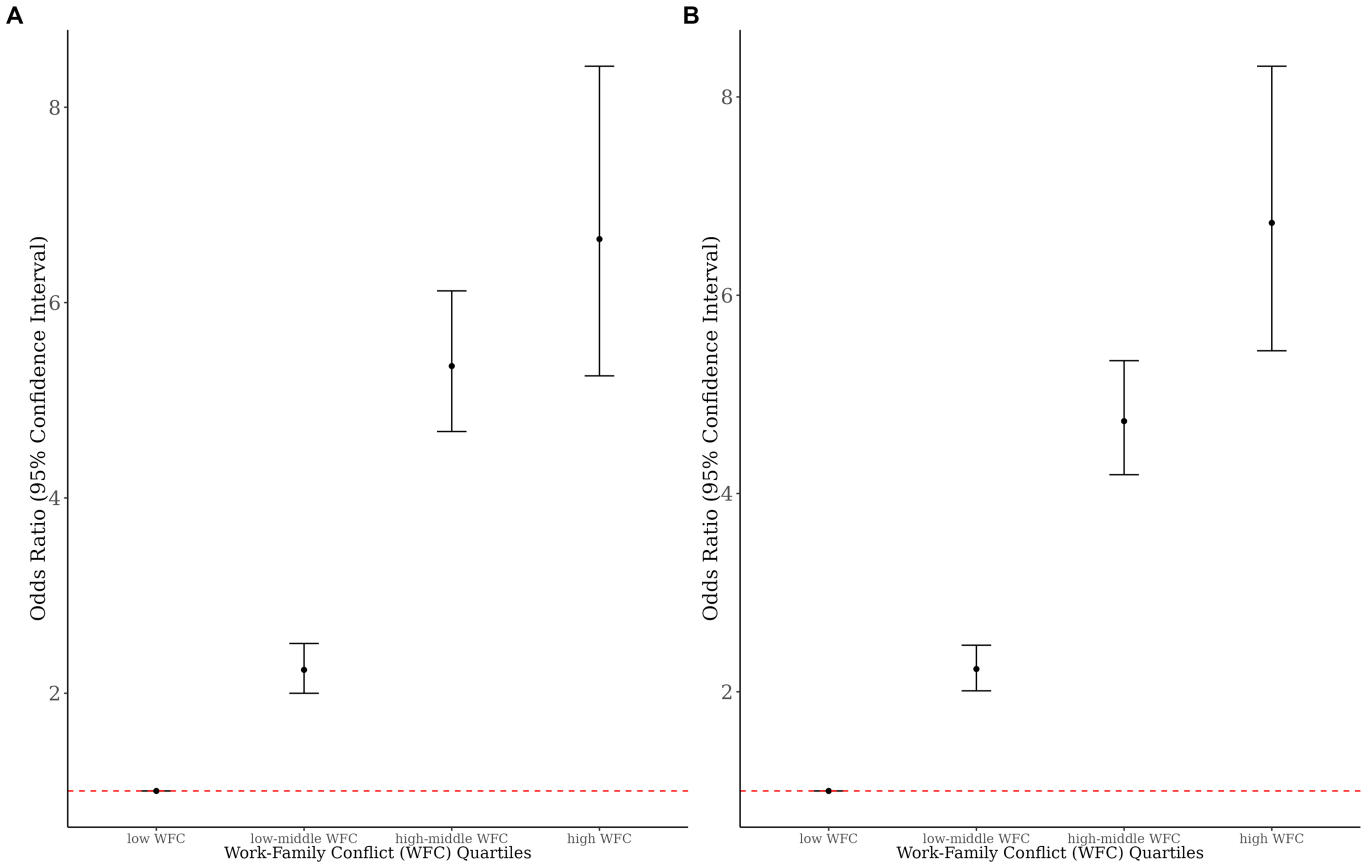
The dose–response relationship of WFC and sleep disturbances by sex **(A)** Male, **(B)** Female.

We found a significant dose–response relationship between WFC quartiles and sleep disturbance in male and female workers (Figure 4). Moreover, the weighted fully adjusted OR (95% CI) of sleep disturbance with WFC in male workers was (2.88 [2.55–3.26]; *p* < 0.001), and the full OR in female workers was (2.75 [2.47–3.07]; *p* < 0.001), which is consistent with the results of the main analysis.

## 4 Discussion

Our cross-sectional study used nationally representative data from Korean workers to highlight the significant association between WFC and sleep disturbance stratified by sex. This association was consistent even after adjusting for covariates, including age, education level, income, working hours, shift work, quick return, and company size. This association displayed a dose-dependent pattern and remained consistent when the survey-weighting approach was employed. Further, the association was significant across all strata within each covariate stratification, indicating a prominent correlation between highly educated workers and those involved in quick returns and larger company sizes, irrespective of sex. Moreover, the interaction analysis demonstrated an additive positive interaction between WFC, quick return to work, and white-collar workers on sleep disturbances (*p* < 0.001), except for white-collar female workers.

Our study highlights a significant correlation between WFC and sleep disturbance in male and female workers, consistent with previous findings (Lallukka et al., 2010; Buxton et al., 2016). Our study revealed a noteworthy association between WFC and sleep disturbance among male workers. This was different from the findings of a previous study, wherein the odds ratio (OR) was 2.56 (95% CI: 1.34–4.87) for male workers and 5.90 (95% CI: [4.16–8.38]) for female (Lallukka et al., 2010). This phenomenon can be attributed to male workers’ changing social roles in contemporary society. Conventionally, male workers had been expected to fulfill the role of breadwinners primarily. Simultaneously, female have had more flexibility to assume various roles, such as mothers, wives, or friends (Heggebø, 2022). However, there has been a notable shift in societal expectations in recent years. In addition to their traditional role as the head of the household, male workers take responsibility for housework and caregiving (Lee and Park, 2021). Consequently, the burden on male workers has doubled. Future studies should use well-structured cohort designs to investigate the impact of this transformation.

In the analysis of sleep disturbance based on WFC within each covariate stratification by sex, individuals with higher levels of education, income, employment in larger companies, and holding white-collar workers exhibited a stronger association. This stronger association was observed between WFC and sleep disturbances. The covariates of education, income, and company size are the primary factors encompassing socioeconomic status (SES; Papadopoulos et al., 2022). Our study found that workers with a higher SES experienced a stronger connection between WFC and reduced sleep disturbance, consistent with previous studies (Kim and Cho, 2020). Individuals with higher SES often have greater ambitions in their career and personal lives, potentially leading to increased internal pressure to achieve these goals (Cochran et al., 2011). Nevertheless, the gap between these ambitions and actual accomplishments can result in severe stress, especially for those with high SES (Sellers and Neighbors, 2008). Stress, strongly linked to sleep disturbances among high-SES workers, may be exacerbated by unfulfilled aspirations and WFC (Cho et al., 2013; Yang B. et al., 2018; Furuichi et al., 2020). Furthermore, in the context of company size, there is an association between working in a large company and various psychosocial factors such as role conflict, quality of management, and sense of purpose in one’s work. Workers within larger companies might experience elevated levels of job-related stress compared to workers in small and medium-sized companies. This is often attributed to the more substantial workload, rapid work pace, the necessity to conceal emotions, and higher emotional demands (Sørensen et al., 2007). Therefore, our study underscores the significance of addressing and managing WFC and associated sleep disturbances, particularly in high-SES populations.

In the RERI analysis, quick returns exhibited an additive interaction with WFC regarding sleep disturbance, irrespective of sex. The potential outcomes of excessive fatigue or time constraints resulting from quick returns may manifest in workers’ sleep disturbances by interfering with their family-related roles (Silva-Costa et al., 2021) or diminishing their daily sleep time while attempting to fulfill these family roles (Crain et al., 2014). As the impact of WFC is more pronounced within the quick return group, individuals with quick returns must consider strategies for mitigating WFC to prevent sleep disturbances. Meanwhile, the importance of the additive interaction between occupation and WFC on sleep disturbance was statistically significant only in male workers. Generally, occupations characterized by physical labor typically maintain regular predetermined working hours. Conversely, white-collar professions often exhibit unrestricted and a propensity for sudden extensions in work hours, which generally has a more significant impact on male workers (Kim and Lee, 2015). Nonetheless, female workers work more in companies offering generous paid family and childcare leave policies or in the public sector, potentially affording them an increased likelihood of experiencing a better working environment than male workers (Cho et al., 2010). These explanations might clarify sex differences in the interactive association between WFC and occupation with sleep disturbance.

To the best of our knowledge, this is the first Korean study to identify risk groups that closely affect the relationship between sleep disturbance and WFC with sex stratification using additive interaction, dose–response analysis, and subgroup analyses. Additionally, contrary to existing studies and hypotheses, the discovery of a high-SES group influencing the association between sleep disturbance and WFC suggests the possibility for additional studies.

Nevertheless, our study had some limitations. First, the cross-sectional study design limited our ability to establish causality and ascertain long-term effects. Future research should consider longitudinal studies and incorporate objective measures of sleep quality to validate and expand our findings. Second, the main variable, “WFC,” was measured through subjective questions. Given the limited nature of the secondary retrospective data, further studies regarding WFC as a main factor in WLB should be conducted with a well-designed prospective cohort. Third, as we conducted the study with employees, the results should be cautiously interpreted when applied to self-employed or contributing family workers. Additionally, our study, limited to Korean workers, may not be generalizable to other ethnic groups. Fourth, several types of sleep disturbances; however, we focused on assessing the presence or absence of sleep disturbance using limited sleep-related questions despite its usefulness as a screening tool. Hence, further studies are necessary to overcome the limited insight into the relationship between WFC and specific sleep disturbances.

## 5 Conclusion

This study highlights the significant association between WFC and sleep disturbance stratified by gender among Korean workers. Our study indicates the significance of managing the relationship between WFC and sleep disturbance, particularly among individuals with higher SES and engaged in quick returns. Our findings also emphasize the importance of appropriate management of WFC in preventing sleep problems, offering a valuable foundation for future research and interventions aimed at enhancing the overall well-being and productivity of the workforce.

## Data availability statement

The datasets presented in this study can be found in online repositories. The names of the repository/repositories and accession number(s) can be found here: https://oshri.kosha.or.kr/oshri/researchField/downWorkingEnvironmentSurvey.do.

## Ethics statement

The study protocol adhered to the ethical principles of the 2013 Declaration of Helsinki and was approved by the Institutional Review Board of Severance Hospital (IRB: 4-2021-1046). The requirement for informed consent was waived owing to the retrospective nature of this study.

## Author contributions

JiL: Conceptualization, Writing – original draft, Data curation, Formal Analysis, Visualization. JO: Conceptualization, Formal Analysis, Visualization, Writing – original draft, Methodology, Project administration, Validation, Writing – review & editing. HP: Writing – original draft, Software. JS: Software, Data curation, Validation, Visualization, Writing – review & editing. JoL: Validation, Writing – review & editing. YK: Validation, Writing – review & editing, Software. BY: Software, Validation, Writing – review & editing, Conceptualization, Funding acquisition, Methodology, Project administration, Resources, Supervision, Writing – original draft.
